# Epidemiological characteristics of respiratory tract infections caused by Mycoplasma pneumoniae in a hospital in Hangzhou, China: A cross-sectional study

**DOI:** 10.1097/MD.0000000000046408

**Published:** 2025-12-19

**Authors:** Hui Zeng, Chunqin Yan, Kailun Cheng, Lihuang Shi, Lijuan Fang, Yidong Wu

**Affiliations:** a Center of Clinical Laboratory, Hangzhou Ninth People’s Hospital, Hangzhou, China.

**Keywords:** epidemiology, MP, pandemic, RTIs

## Abstract

Mycoplasma pneumoniae (MP), a major pathogen that causes respiratory tract infections (RTIs) in humans, represents a predominant form of community-acquired pneumonia (CAP) especially in children. The high infection rate and Macrolides resistance (MR) rate in China poses a significant threat to the public health. We retrospectively analyzed data of individuals from July 2018 to August 2024 who visited Hangzhou Ninth People`s Hospital due to RTIs and did a fluorescence quantitative PCR (qPCR) test for MP. A total of 25,980 individuals were included, among which 6020 (23.17%) were infected with MP. MP infections were more prevalent in late summer and autumn, with the most in October (1022), followed by November (995). The infection number dropped significantly in winter and spring, with the least in March (170), followed by February (192) and April (192). The infections in both the pre-COVID-19 pandemic year (2019) and post-COVID-19 pandemic year (2023) were much higher than the years during COVID-19 pandemic (2020, 2021, 2022). Non-pharmaceutical interventions significantly impact the spread of MP. MP infection mainly infected preschool and lower grade school aged children.1 to 5 and 6 to 10 years children accounted for the most MP cases, with 2177 and 2751 cases respectively. Considering MRMP, the results showed a positive rate of 77.66%, with the highest in 4 to 18 years children (81.07%) and lowest in <1 year old children (60.00%). There was no significant difference in neither MP positive rate nor MRMP positive rate between male and female.

## 1. Introduction

Mycoplasma pneumoniae (MP) is presently the smallest prokaryotic microorganism capable of independent survival without a host cell found in nature,^[1]^ and concurrently serves as the leading etiological agent responsible for pediatric community-acquired pneumonia (CAP).^[2]^ It was first isolated in 1944 by Eaton et al^[3]^ from sputum sample of a patient having primary atypical pneumonia and was naturally called as Eaton agent. Later in 1963 Chanock et al,^[4]^ proposed nomenclature for atypical pneumonia organism and designated Eaton agent as M. pneumoniae. It is transmitted via contacting and inhaling with aerosols or droplets discharged by infected people. After inhalation, MP adheres to the cilated cells of epithelium lining of the respiratory tract with an attachment organelle and causes a series of subsequent pathological changes. The pathogenic mechanism of MP predominantly encompasses 5 aspects: adherence, immune evasion, inflammation injury, cytotoxicity, and gliding motility.^[5]^ MP causes respiratory tract infections (RTIs) with diverse manifestations mainly limited to the respiratory system. Among which tracheobronchitis is the most common manifestation, and pneumonia is the most clinically important illness. In addition to pulmonary manifestations, MP infection can also cause extrapulmonary manifestations, such as cardiovascular (myositis,),^[6]^ dermatological (Stevens–Johnson syndrome, small-vessel cutaneous vasculitis),^[7]^ hematopoietic system (Autoimmune hemolytic anemia, hemophagocytic syndrome),^[8]^ musculoskeletal (arthritis)^[9]^ and neurologic (encephalitis, myelitis, meningitis),^[10]^ as well as other organ involvement.^[11,12]^ The severity of the infection can range from mild, self-limiting symptoms to severe death causing. MP occurs endemically worldwide, and its epidemic occurs periodically. The incidence of MP has been reported to increase significantly in recent years.

To better understanding the epidemiological characteristics of MP infection and provide evidence for the clinical diagnosis, treatment and prevention and control strategies making in the local region, we did this study.

## 2. Materials and methods

### 2.1. Data source and ethical concerns

All data were retrospectively collected from electronic laboratory system of Hangzhou Ninth People`s Hospital in Hangzhou, Zhejiang province. All patients suspected of MP infection who had nasopharyngeal or oropharyngeal swabs collected for an MP nucleic acid test were included in the study. Data covered the period from July 2018 to August 2024. This study is based on the completely retrospective analysis of anonymized data. Ethic approval has been granted by the Medical Ethics Committee of Hangzhou Ninth People`s Hospital.

### 2.2. Laboratory assay

During July 1, 2018 to August 3, 2023, MP and macrolide-resistant isolates were detected by a commercial diagnostic kit (Mole Bioscience, Taizhou, China). The kit uses polymerase chain reaction (PCR) combined with Taqman fluorescent probe technology to detect MP nucleic acid and its mutation sites in human throat swab specimens. Primer probes labeled with VIC fluorescence were designed for the conserved region of P1 gene to detect MP. Primer probes labeled with FAM fluorescence were designed to detect A2063G and A2064G mutations at the 23S rRNA gene mutation site. During August, 2023 to August 31, 2024, MP was detected by a commercial 6 respiratory pathogen nucleic acid testing kit (Sansure Bioteck Ink. Changsha, China).

### 2.3. Statistical analysis

We performed a descriptive analysis on the data. In this study one year was defined from March to February of the following year, which allows each year contains all 4 seasons. The database was built with Microsoft Excel software, and IBM SPSS statistics 19 software was used for data extraction and description. The peak period was defined as the period with monthly MP positive rate over annual rate. The Pearson chi-square test was employed to compare detection rates across groups. For pairwise comparisons across multiple groups, the Bonferroni method was applied for correction. A two-tailed test was performed with a significance level of α = 0.05.

## 3. Results

### 3.1. Prevalence characteristics of MP

#### 3.1.1. Study population and an overview of MP infection

**Figure 1.**
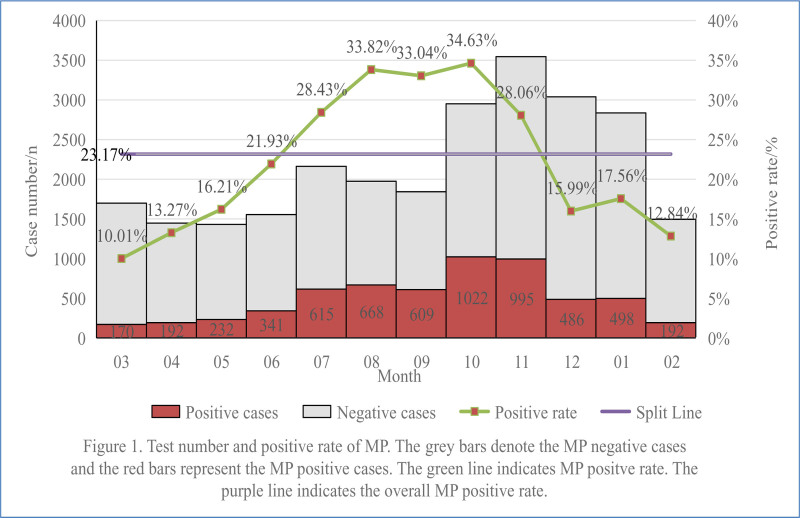
Test number and positive rate of MP. The gray bars denote MP negative cases and the red bars represent the MP positive cases. The green line indicates MP positive rate. The purple line indicates the overall MP positive rate. MP = Mycoplasma pneumoniae.

From July 1, 2018, to August 31, 2024, a total of 25,980 patients with ARTIs were tested PCR as part of the MP diagnosis. The median age was 60 months (interquartile range 36–120 months). MP was detected in 6020 patients, which took 23.17% of the total. Median age of MP positive patients was 72 months (interquartile range 48–108 months). ARTIs were more prevalent in late autumn and early winter, with a monthly incidence of around 3000 cases in these months and the highest was in November with a total of 3546 respiratory infections (Fig. 1). The number of ARTIs in every other month was at about 1500 cases per month. However, MP infections mainly occur in late summer and autumn, with the highest rate of 31.49% in autumn, followed by summer (28.53%), winter (15.96%) and the lowest in spring (12.98%) (Table 1). MP positive rates increased significantly from July, peaked at 34.63% in October. The peak period lasted for 5 months, starting in July and ending in December. MP cases were highest in October and November, with 1022 and 995 respectively (Fig. 1).

**Table 1:** Distribution of MP.

	Total	Pos. cases	Neg. cases	Pos. rate (%)	Pos. ratio (%)	χ^2^	*P*
Overall	25,980	6020	19,960	23.17	100.00		
Gender							
Male	13,575	3140	10,435	23.13	52.16	0.027	.871
Female	12,405	2880	9525	23.22	47.84		
Season							
Spring	4577	594	3983	12.98	9.87	898.271	<.001*
Summer	5693	1624	4069	28.53	26.98		
Autumn	8340	2626	5714	31.49	43.62		
Winter	7370	1176	6194	15.96	19.53		
Year							
2018–	1591	215	1376	13.51	3.57	1315.657	<.001**
2019	3124	751	2373	24.04	12.48		
2020	972	16	956	1.65	0.27		
2021	1560	65	1495	4.17	1.08		
2022	2008	336	1672	16.73	5.58		
2023	12,319	3908	8411	31.72	64.92		
2024-	4406	729	3677	16.55	12.11		
Age							
<1	2019	111	1908	5.50	1.84	2757.178	<.001
1–5	11,727	2177	9550	18.56	36.16		
6–10	6080	2751	3329	45.25	45.70		
11–15	1210	358	852	29.59	5.95		
16–20	152	27	125	17.76	0.45		
21–25	172	52	120	30.23	0.86		
26–30	367	80	287	21.80	1.33		
31–35	508	154	354	30.31	2.56		
36–40	288	75	213	26.04	1.25		
41–45	175	26	149	14.86	0.43		
46–50	195	15	180	7.69	0.25		
51–55	304	39	265	12.83	0.65		
56–60	367	40	327	10.90	0.66		
61–65	377	33	344	8.75	0.55		
66–70	450	22	428	4.89	0.37		
71–75	426	19	407	4.46	0.32		
76–80	415	19	396	4.58	0.32		
>80	748	22	726	2.94	0.37		

Positive rate is taken as a proportion of total tests.

Positive ratio is taken as a proportion of overall positive cases.

MP = Mycoplasma pneumoniae.

* The *P*-values are all <.001, when comparing each pair of different seasons.

** For any comparison between 2 yr within the period from 2019 to 2023, the *P*-values are all <.001.

#### 3.1.2. Annual analyses on MP infection

**Figure 2.**
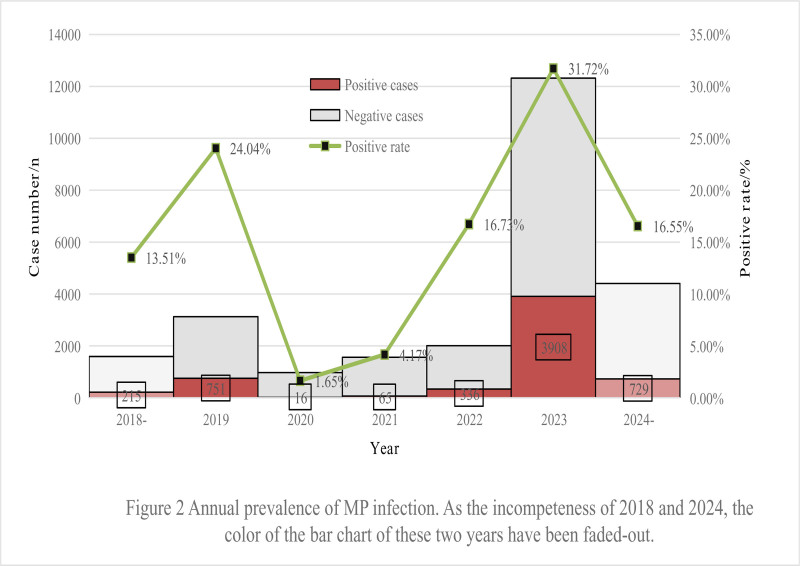
Annual prevalence of MP infection. As the incompleteness of 2018 and 2024, the color of the bar chart of these 2 yr have been faded-out. MP = Mycoplasma pneumoniae.

Due to incomplete data in 2018 and 2024, we mainly analyze the data from 2019 to 2023 in the annual analysis. In order to ensure the integrity of all 4 seasons, we will start a whole year from March of one year to February of the following year. Annual cases and the positive rate were shown in Table 1. Both the number of the ARTIs patients and the positive rates of MP in pre-COVID-19 pandemic (2019) and post-COVID-19 pandemic (2023) were much higher than those during COVID-19 epidemic (2020, 2021, and 2022), especially in 2023 which were particularly higher than those during 2020 to 2021. Before the pandemic, MP positive rate was 24.04% (751/3124) in 2019. During the pandemic, the rate plummeted to 1.65% (16/972) in 2020 and 4.17% (65/1560) in 2021 (Table 1). Although not obvious, an increased positive rate was already observed in 2022 with a positive rate of 16.73% (336/2008), then following a surge to 31.72% (3908/12,319) in 2023 (Fig. 2).

#### 3.1.3. Monthly analyses on MP infection

**Figure 3.**
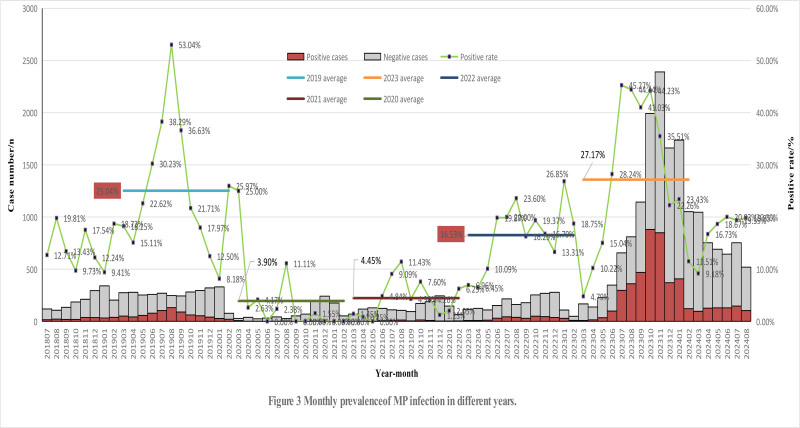
Monthly prevalence of MP infection in different years. MP = Mycoplasma pneumoniae.

We further performed a monthly MP infection analysis and found that the monthly distribution of MP varied from year to year in the years we analyzed. The monthly cases for the total study period are shown in Figure 3. Before the outbreak of COVID-19, the distribution of respiratory diseases suspected to be infected with MP was relatively average every month. There was one peak period covered summer and early autumn and lasted for 4 months (June–September), with the highest MP positive rate of 53.04% and also the most MP cases in August in 2019. During COVID-19 pandemic, not only are infections of MP rare, but also respiratory diseases are greatly reduced under the strict pandemic prevention policies, especially in 2020. However, with the ease of pandemic-related policies, respiratory diseases, including MP, were gradually on the rise. By 2022, the number of MP cases rebounded. From June 2022 to January 2023, the positive rate of MP showed a high level, higher than the annual average, except for December 2022, where the number of cases was still relatively high. For 2022, the highest MP positive rate occurred in January 2023, with the most MP cases in October and November 2022. Then with the comprehensive opening of the pandemic in December 2022, during 2023 year the number of MP cases began to increase from June 2023, reaching over 800 cases in both October and November, and then began to decline significantly to 121 cases in February 2024. The peak period of 2023 lasted 6 months (June–November). Although the number of cases in 2022 was far from comparable to that of 2023, the prevalence of MP showed a similar seasonal distribution, the number of MP positive cases both started to rise in June, peaked in October, and remained high until the end of winter. After a slight decline in March 2024, there was a slight rebound in April, and until August, the infection of MP remained at a stable level with over 100 MP positive cases and an MP positive rate of over 16%. The epidemiological trend of MP can be seen in the Figure 3.

#### 3.1.4. Analyses on MP infection by seasonality

MP positive rates in summer (40.13%) and autumn (24.79%) of 2019 was higher than those in winter (12.66%) and spring (18.53%), but unlike the overall distribution where autumn was slightly higher than summer, the infection rate in summer of 2019 was much higher than that in autumn. Although the infection rates during the pandemic were low, the seasonal distribution characteristics were also characterized by the highest positive rates in summer, which were 8.26% and 21.06% in 2021 and 2022, respectively. In 2020, the highest rate was 10.00% in winter, followed by 4.00% in summer. Although there are slight seasonal differences in the peak period of MP from 2019 to 2023, they all occur in summer and autumn. Except for 2020, the highest rate occurred in spring may be due to subsequent epidemic prevention and control strategies leading to a decrease in infections in subsequent seasons and a relative increase in spring (Table S1, Supplemental Digital Content, https://links.lww.com/MD/Q839).

#### 3.1.5. Analyses on MP infection by age stratification

Except for those under one year old and those over 80 years old, all other patients were grouped in groups of every 5 years old. And the patients were divided into 18 groups. As shown in Table 1.

Distribution of the total MP patient population based on age showed the highest proportion of positive detections within the 1 to 5 and 6 to 10 age groups [2177/6020 (36.16%), 2751/6020 (45.70%), respectively] followed by 11 to 15 years [358/6020 (5.95%)]. Among adults, the 31 to 35 age group has the highest proportion, accounting for 2.56% of the overall population. Next are the 26 to 30 age group and the 36 to 40 age group, respectively. The distribution of MP positive cases and the overall population distribution from 2019 to 2024 remained consistent, with children aged 1 to 10 accounting for the highest proportion. However, the phenomenon of adults aged 26 to 40 accounting for the highest proportion was reflected in 2019, 2022, 2023, and 2024, while it was not significant in 2020 and 2021 (Table S2, Supplemental Digital Content, https://links.lww.com/MD/Q839).

#### 3.1.6. Analyses on MP infection by gender

Among the 25,280 included cases, 13,575 were male, and 12,405 were female. The positive rates of MP were 23.13% in male and 23.22% in female. Among both patients with respiratory symptoms and patients with MP infection, there were more males than females, but the difference was not statistically significant. There was no gender difference in the positivity rate of MP infection in each year from 2018 to 2024 (Table S3, Supplemental Digital Content, https://links.lww.com/MD/Q839).

#### 3.1.7. Prevalence characteristics of MRMP

A total of 1625 MP samples had been obtained to evaluated the prevalence of macrolide resistance. The overall positive rate of MRMP test was 77.66%. The positive rate of MRMP between genders showed 76.83% resistance in males and 78.61% in females, with no significant difference. Then the enrolled individuals were divided into 4 groups by age: infancy (<1 age group), 1 to 8 age group, 9 to 18 age group and adults (>18 years old) based on antibiotics use for comparative analysis of MRMP. The results showed that the resistant rate in infancy was 60.00%, lower than 78.88% in 1 to 8 age group and 80.47% in 9 to 18 age group, then decreased to 69.59% in adults. The differences among different age groups were statistically significant. Annual analysis showed that the highest resistant rate is 82.23% in 2023-, the lowest is 56.25% in 2020 when the MP positive number was also the lowest during the analyzing period. The remaining years were 61.86% in 2018-, 80.29% in 2019, 69.23% in 2021, 81.25% in 2022 (Table 2).

**Table 2:** The prevalence characteristics of MRMP.

MRMP	Total	Resistant	Sensitive	Resistant rate	χ^2^	*P*
Total	1625	1262	363	77.66%		
Gender						
Male	863	663	200	76.83%	0.742	.404
Female	762	599	163	78.61%		
Season						
Spring	243	180	63	74.07%	16.476	.001*
Summer	677	559	118	82.57%		
Autumn	427	320	107	74.94%		
Winter	278	203	75	73.02%		
Age						
<1	50	30	20	60.00%	16.55	.001**
1–8	1212	956	256	78.88%		
9–18	215	173	42	80.47%		
>18	148	103	45	69.59%		
Year						
2018-	215	133	82	61.86%	46.238	<.001***
2019	751	603	148	80.29%		
2020	16	9	7	56.25%		
2021	65	45	20	69.23%		
2022	336	273	63	81.25%		
2023-	242	199	43	82.23%		

MP = Mycoplasma pneumoniae, MRMP = macrolide-resistant MP.

* Summer versus Spring: *P* = .006; Summer versus Winter: *P* = .001; Summer versus Autumn: *P* = .003.

** <1 versus 1–8: *P* = .003; <1 versus 9–18: *P* = .005; >18 versus 1–8: *P* = .015; >18 versus 9–18: *P* = .024.

*** 2019 versus 2020: *P* = .027; 2019 versus 2021: *P* = .039; 2022 versus 2019: *P* = .023; 2022 versus 2021: *P* = .043.

## 4. Discussion

MP is a cell-wall lacking bacterial pathogen that causes human respiratory infection. It grows slowly and may cause a slow and continuous course of illness, cough caused by MP infection can even last for one to several months. Even though some MP infections do not cause symptoms, they increase the susceptibility of infected individuals to other pathogens. MP infections occur sporadically in different regions year-round, but tend to be more common in summer and late autumn.^[13]^ Our data show that MP infection is more common in late summer and autumn, with the most monthly cases in October. Based on a 9-year period study on children, Japanese researchers have revealed that MP infection was positively correlated with average temperature and relative humidity.^[14]^ Another study in 2017 in Hangzhou also showed a positive correlation between temperature and MP infection rate and they also found a significant lag effect of temperature on the MP infection rate,^[15]^ which may help to explain the increased cases in late summer and autumn.

In accordance with most previous studies,^[16,17]^ MP infection decreased remarkably during the COVID-19 pandemic and rebounded in 2023. Though MP infections decreased remarkably in both the number of positive cases and MP positive rate during COVID-19 pandemic (with 1.65% positive rate in 2020, 4.17% in 2021 and 16.73% in 2022) in our study, Chen et al^[18]^ exhibit a relatively high positive rate during 2020 to 2022 with positive rates of 24.64%, 41.86% and 38.08% respectively in a study based on 66,488 pediatric patients with respiratory tract infection in Anhui. As the geographical location of Anhui province is adjacent to Zhejiang province, the climate difference between the 2 regions is not significant. Therefore, we speculate that there are 3 reasons for the infection difference. Firstly, the research subjects are different, with the Gao et al.`s being children aged 0 to 14, while our study subjects mainly cover the entire age group. Secondly, the detection methods are different. Antibody titer in serum is measured in Gao et al’s study, while nucleic acid is detected in throat swab in our study. Finally, we have different definition of the year division, with each year in Gao`s study from January to December of the year, while we start from March of the year to February of the following year. Coincidentally, another study^[19]^ in Henan Province based on data from 2018 to 2021 shows that although the positive rate of MP in 2020 (1019 cases, 15.01% positive rate) and 2021 (1333, 18.08%) decreased compared with that in 2019 (2619, 22.46%), the positive cases and positive rate did not show such a significant decline as in our study, though their research detection method is also PCR. The positive rates show an increase compared to those in 2018 (1288, 14.72%). The difference may be contributed to the regional differences, different enrolled patients and different definitions of years. There are differences in climate and living habits between northern China and southern China, differences in infection rate and seasonality of MP exhibit correspondently.^[20–23]^ They studied the MP infection in children with CAP and defined a year from January to December.

Throughout the entire COVID-19 pandemic period, respiratory infectious diseases have been greatly reduced under the prevention and control strategies. China has been facing several outbreaks of respiratory diseases since the COVID-19 related restriction has been eased. And most of the resurgent respiratory diseases outbroke during the end of the 2021 and the end of 2022. And influenza and respiratory syncytial virus drove most spikes in illness. In a previous study,^[24]^ we detected a pronounced reemergence in influenza infection as early as the end of 2021, reaching a peak at the beginning of 2022, and then reaching a higher peak during the summer of 2022 in our hospital. Considering MP, though at low level, an increase in MP infection was noted in 2022. The remarkable upsurge of MP infection occurred in the summer and autumn of 2023. It is obvious that strict prevention and control strategies during the pandemic can effectively block the spread of the respiratory infectious diseases. However, with the gradual relaxation of prevention and control strategies and the waning herd immunity, there has been a significant rebound in respiratory diseases. As resurgence of many other respiratory pathogens was gradually observed in early 2021, resurgence of MP can be hard to solely attribute to control strategies relaxation and waning herd immunity. Previous data indicated that MP epidemic occurs every of 3 to 7 years and lasts 2 years. The most recent epidemic is recorded in the late 2019 globally,^[25]^ our data also show an epidemic in 2019. Hang Li and his colleagues’ report^[26]^ suggested that MP could have caused outbreaks across the country as early as 2020 based on phylogenetic analysis. We postulate that the sudden outbreak of COVID-19 broke the MP epidemic that could have continued into 2020. The delayed reemergence of MP may partly contribute to the transient herd immunity from last epidemic in 2019 to 2020.

Considering the effect of gender on MP susceptibility, our data show no difference. Although most studies have shown little effect of gender on MP infection,^[17,20]^ a study^[27]^ conducted in Hangzhou on data from 2007 to 2009 indicated a higher positive rate in female, which is in line with a recent study from Anhui.^[18]^ The difference between different studies may contribute to many factors, including the study population, detection methods, research region, and so on. The 2 studies both focus on MP infection on children, whereas we study the entire age groups. And coinfection with other pathogens may also affect the different MP infection in different gender. MP infection is higher in male patients than female when coinfected with Adenovirus.^[28]^ Coinfection may further complex the situation.

Similar to previous report,^[20]^ the positive rate and positive cases of children are much higher than those of adults. 1 to 5, 6 to 10, 11 to 15 age groups account for over 80% MP cases. And the highest rate is found in 6 to 10 age group which is in consistent with most studies.^[20,29]^ some studies^[18,20]^ exhibit a positive correlation between MP positive rate and age in children. However, our study does not show such a correlation. The MP positive rate shows an increasing trend with age in the <1 year old, 1 to 5 years old, and 6 to 10 years old groups, but began to decrease from 11 to 15 years old group. The difference may be attributed to the different age stratification. Other studies group a >7 years old group. From our results, we can see that age group of 5 to 10 has the highest positive rate and the most MP cases. The number of MP cases is more than 7 times that of the 11 to 15 age group, and the positive rate being more than 1.5 times higher.

Macrolide has been used as the first-line medicine for treatment of MP infection for a long time. However, with the widespread empirical use of macrolide, resistance gradually develops. Macrolide-resistant MP (MRMP) was first isolated from clinical patients in 2000 in Japan, then successively reported in other countries, with different rates in different regions, usually with a high level in Asian countries and a low level in European countries.^[30–33]^ In China, the rate differs in different regions, ranging from 20 to 100%.^[33–35]^ The resistance of MP to macrolide has been confirmed to be attribute to the point mutations in the V region of 23S rRNA gene, and the mutation at position A2063G is the most frequent position, followed by A2064G, others are rare.^[35–37]^ Our study shows a 77.66% resistant rate and macrolide resistance are independent of gender. However, the MRMP rate differs in different age groups. Okazaki et al^[38]^ reported macrolide susceptible MP may convert to macrolide resistant MP in the presence of macrolide exposure. The guidelines for the diagnosis and treatment of Mycoplasma Pneumoniae Pneumonia (2023 edition) in children^[39]^ state that macrolides are the preferred choice for anti-MP treatment. New tetracyclines as alternative drugs are only suitable for children over 8 years old, and full evaluation is required for children under 8 years old. Quinolones are alternative drugs for Mycoplasma Pneumoniae Pneumonia, but their use in children under 18 years old is beyond the instructions and also requires full evaluation. MP infected individuals over 18 years old have more options for antibiotics, while those under 1 year old are more cautious when taking medication. For individuals between 1 to 18 years age, they rely more on macrolides for treatment in younger children (<8 years old), while for older children (>8 years old), although there are more drug options for treatment, macrolides are still the preferred choice. And guardian may be more casual in their use of drugs for older children than for young children.

There are several limitations to this study. Firstly, it is a single-site based retrospective analysis. Secondly, it is limited by the limited period and limited data. Although we cover periods before, during and after the entire course of COVID-19, periods before and after COVID-19 are not long enough. The data of 2018, 2024 and MRMP are incomplete.

Although an organized surveillance program may be more important for assessing the true impact of MP on public health, data analysis from our study can also provide some insight into the understanding of the prevalence of MP and MRMP infection and provide evidences for preventing and controlling. Our study also has some advantages, unlike other studies that either included CAP patients or upper respiratory tract patients, or only included pediatric patients and excluded adults, we conduct a systematic exploration of MP infection and give a comprehensive insight into the infection. We include all age groups of patients with respiratory infections suspected MP infection. In addition, PCR is a rapid, accurate method for early diagnosis of MP, and all patients we included were tested for their throat swabs using PCR test.

In conclusion, ARIs exhibit a season pattern, mostly with higher incidence during autumn and winter, but high MP infection is usually in summer and autumn. MP infection characteristics have not changed much before and post the pandemic. The striking increase of MP in 2023 may largely attribute to the dual effects of waning herd immunity and cyclic characteristic of MP epidemic. The deterioration of the air environment, emergence of new viruses such as COVID-19, changes of the disease prevention and control strategies and the extensive use of antibiotics have complicated the prevalence, the manifestation and the treatment of MP infection. A continuous surveillance of MP will surely help to better understanding the characteristics of MP, thereby contributing to MP infection prevention and control.

## Author contributions

**Writing – original draft**: Hui Zeng, Chunqin Yan.

**Formal analysis:** Hui Zeng, Kailun Cheng.

**Tables and figures**: Lihuang Shi, Hui Zeng.

**Experimental facility**: Lijuan Fang.

**Supervision**: Yidong Wu.

**Conceptualization**: Yidong Wu.

**Review**: Yidong Wu.

## Supplementary Material


