# Supplementary material for: Epidemiological characteristics of respiratory tract infections caused by Mycoplasma pneumoniae in a hospital in Hangzhou, China: A cross-sectional study
Source: Medicine (Baltimore). 2025 Dec 19;104(51):e46408. doi: 10.1097/MD.0000000000046408 (PMC12727402; doi:10.1097/MD.0000000000046408)
Supplement: Supplementary file 1 [file medi-104-e46408-s001.docx]

| Supplementary Table 1 Seasonality difference in the prevalence of MP infection. | | | | | | | | |
| --- | --- | --- | --- | --- | --- | --- | --- | --- |
| Year  Season | Overall | *2018*- | 2019 | 2020 | 2021 | 2022 | 2023 | *2024-* |
| spring | 594/4577 (12.98%) | / | 149/804 (18.53%) | 9/90 (10.00%) | 2/315 (0.63%) | 27/348 (7.76%) | 56/530  (10.57%) | 351/2490 (14.10%) |
| summer | 1624/5693 (28.53%) | 36/224 (16.07%) | 312/774  (40.31%) | 4/100 (4.00%) | 28/339 (8.26%) | 111/527 (21.06%) | 755/1813 (41.64%) | 378/1916 (19.73%) |
| autumn | 2626/8340 (31.49%) | 73/530 (13.77%) | 203/819 (24.79%) | 3/314 (0.96%) | 25/452 (5.53%) | 123/699 (17.60%) | 2199/5526 (39.79%) | / |
| winter | 1176/7370 (15.96%) | 106/83 (12.66%) | 87/727 (11.97%) | 0/468 (0.00%) | 10/454 (2.20%) | 75/434 (17.28%) | 898/4450 (20.18%) | / |

| Supplementary Table 2 Age difference of MP infection in different years. | | | | | | | | | | |
| --- | --- | --- | --- | --- | --- | --- | --- | --- | --- | --- |
| **Age group** | **Total MP test** | **total positive** | **Pos.** | **MP Pos. ratio** | **MP Pos. rate** | **2018- MP test** | **total positive** | **Pos.** | **MP Pos. ratio** | **MP Pos. rate** |
| **<1** | 2019 | 6020 | 111 | 1.84% | 5.50% | 318 | 215 | 15 | 6.98% | 4.72% |
| **1-5** | 11727 | 6020 | 2177 | 36.16% | 18.56% | 1015 | 215 | 116 | 53.95% | 11.43% |
| **6-10** | 6080 | 6020 | 2751 | 45.70% | 45.25% | 189 | 215 | 74 | 34.42% | 39.15% |
| **11-15** | 1210 | 6020 | 358 | 5.95% | 29.59% | 20 | 215 | 7 | 3.26% | 35.00% |
| **16-20** | 152 | 6020 | 27 | 0.45% | 17.76% | 0 | 215 | 0 | 0.00% | 0.00% |
| **21-25** | 172 | 6020 | 52 | 0.86% | 30.23% | 2 | 215 | 1 | 0.47% | 50.00% |
| **26-30** | 367 | 6020 | 80 | 1.33% | 21.80% | 6 | 215 | 1 | 0.47% | 16.67% |
| **31-35** | 508 | 6020 | 154 | 2.56% | 30.31% | 1 | 215 | 0 | 0.00% | 0.00% |
| **36-40** | 288 | 6020 | 75 | 1.25% | 26.04% | 0 | 215 | 0 | 0.00% | 0.00% |
| **41-45** | 175 | 6020 | 26 | 0.43% | 14.86% | 1 | 215 | 0 | 0.00% | 0.00% |
| **46-50** | 195 | 6020 | 15 | 0.25% | 7.69% | 6 | 215 | 0 | 0.00% | 0.00% |
| **51-55** | 304 | 6020 | 39 | 0.65% | 12.83% | 5 | 215 | 1 | 0.47% | 20.00% |
| **56-60** | 367 | 6020 | 40 | 0.66% | 10.90% | 9 | 215 | 0 | 0.00% | 0.00% |
| **61-65** | 377 | 6020 | 33 | 0.55% | 8.75% | 3 | 215 | 0 | 0.00% | 0.00% |
| **66-70** | 450 | 6020 | 22 | 0.37% | 4.89% | 7 | 215 | 0 | 0.00% | 0.00% |
| **71-75** | 426 | 6020 | 19 | 0.32% | 4.46% | 4 | 215 | 0 | 0.00% | 0.00% |
| **76-80** | 415 | 6020 | 19 | 0.32% | 4.58% | 3 | 215 | 0 | 0.00% | 0.00% |
| **>80** | 748 | 6020 | 22 | 0.37% | 2.94% | 2 | 215 | 0 | 0.00% | 0.00% |
| **Age group** | **2019 MP test** | **total positive** | **Pos.** | **MP Pos. ratio** | **MP Pos. rate** | **2020 MP test** | **total positive** | **Pos.** | **MP Pos. ratio** | **MP Pos. rate** |
| <1 | 357 | 751 | 26 | 3.46% | 7.28% | 142 | 16 | 1 | 6.25% | 0.70% |
| 1-5 | 1706 | 751 | 394 | 52.46% | 23.09% | 573 | 16 | 3 | 18.75% | 0.52% |
| 6-10 | 467 | 751 | 200 | 26.63% | 42.83% | 62 | 16 | 7 | 43.75% | 11.29% |
| 11-15 | 90 | 751 | 32 | 4.26% | 35.56% | 12 | 16 | 1 | 6.25% | 8.33% |
| 16-20 | 10 | 751 | 2 | 0.27% | 20.00% | 3 | 16 | 0 | 0.00% | 0.00% |
| 21-25 | 16 | 751 | 8 | 1.07% | 50.00% | 3 | 16 | 1 | 6.25% | 33.33% |
| 26-30 | 42 | 751 | 12 | 1.60% | 28.57% | 9 | 16 | 0 | 0.00% | 0.00% |
| 31-35 | 65 | 751 | 22 | 2.93% | 33.85% | 5 | 16 | 0 | 0.00% | 0.00% |
| 36-40 | 17 | 751 | 8 | 1.07% | 47.06% | 2 | 16 | 0 | 0.00% | 0.00% |
| 41-45 | 24 | 751 | 5 | 0.67% | 20.83% | 3 | 16 | 0 | 0.00% | 0.00% |
| 46-50 | 26 | 751 | 4 | 0.53% | 15.38% | 18 | 16 | 0 | 0.00% | 0.00% |
| 51-55 | 42 | 751 | 8 | 1.07% | 19.05% | 23 | 16 | 0 | 0.00% | 0.00% |
| 56-60 | 55 | 751 | 6 | 0.80% | 10.91% | 19 | 16 | 1 | 6.25% | 5.26% |
| 61-65 | 57 | 751 | 10 | 1.33% | 17.54% | 20 | 16 | 1 | 6.25% | 5.00% |
| 66-70 | 45 | 751 | 5 | 0.67% | 11.11% | 24 | 16 | 0 | 0.00% | 0.00% |
| 71-75 | 40 | 751 | 5 | 0.67% | 12.50% | 20 | 16 | 0 | 0.00% | 0.00% |
| 76-80 | 25 | 751 | 3 | 0.40% | 12.00% | 12 | 16 | 1 | 6.25% | 8.33% |
| >80 | 40 | 751 | 1 | 0.13% | 2.50% | 22 | 16 | 0 | 0.00% | 0.00% |
| **Age group** | **2021 MP test** | **total positive** | **Pos.** | **MP Pos. ratio** | **MP Pos. rate** | **2022 MP test** | **total positive** | **Pos.** | **MP Pos. ratio** | **MP Pos. rate** |
| **<1** | 227 | 65 | 3 | 4.62% | 1.32% | 196 | 336 | 5 | 1.49% | 2.55% |
| **1-5** | 983 | 65 | 30 | 46.15% | 3.05% | 1208 | 336 | 140 | 41.67% | 11.59% |
| **6-10** | 160 | 65 | 28 | 43.08% | 17.50% | 366 | 336 | 143 | 42.56% | 39.07% |
| **11-15** | 22 | 65 | 1 | 1.54% | 4.55% | 68 | 336 | 14 | 4.17% | 20.59% |
| **16-20** | 1 | 65 | 0 | 0.00% | 0.00% | 3 | 336 | 1 | 0.30% | 33.33% |
| **21-25** | 4 | 65 | 0 | 0.00% | 0.00% | 7 | 336 | 3 | 0.89% | 42.86% |
| **26-30** | 11 | 65 | 0 | 0.00% | 0.00% | 11 | 336 | 5 | 1.49% | 45.45% |
| **31-35** | 12 | 65 | 0 | 0.00% | 0.00% | 22 | 336 | 13 | 3.87% | 59.09% |
| **36-40** | 5 | 65 | 0 | 0.00% | 0.00% | 18 | 336 | 7 | 2.08% | 38.89% |
| **41-45** | 5 | 65 | 0 | 0.00% | 0.00% | 8 | 336 | 1 | 0.30% | 12.50% |
| **46-50** | 11 | 65 | 0 | 0.00% | 0.00% | 12 | 336 | 1 | 0.30% | 8.33% |
| **51-55** | 11 | 65 | 1 | 1.54% | 9.09% | 11 | 336 | 2 | 0.60% | 18.18% |
| **56-60** | 17 | 65 | 0 | 0.00% | 0.00% | 15 | 336 | 0 | 0.00% | 0.00% |
| **61-65** | 18 | 65 | 1 | 1.54% | 5.56% | 17 | 336 | 0 | 0.00% | 0.00% |
| **66-70** | 22 | 65 | 1 | 1.54% | 4.55% | 14 | 336 | 1 | 0.30% | 7.14% |
| **71-75** | 11 | 65 | 0 | 0.00% | 0.00% | 7 | 336 | 0 | 0.00% | 0.00% |
| **76-80** | 14 | 65 | 0 | 0.00% | 0.00% | 10 | 336 | 0 | 0.00% | 0.00% |
| **>80** | 26 | 65 | 0 | 0.00% | 0.00% | 15 | 336 | 0 | 0.00% | 0.00% |
| **Age group** | **2023 MP test** | **total positive** | **Pos.** | **MP Pos. ratio** | **MP Pos. rate** | **2024- MP test** | **total positive** | **Pos.** | **MP Pos. ratio** | **MP Pos. rate** |
| <1 | 469 | 3908 | 48 | 1.23% | 10.23% | 310 | 729 | 13 | 1.78% | 4.19% |
| 1-5 | 4672 | 3908 | 1242 | 31.78% | 26.58% | 1570 | 729 | 252 | 34.57% | 16.05% |
| 6-10 | 3821 | 3908 | 1988 | 50.87% | 52.03% | 1015 | 729 | 311 | 42.66% | 30.64% |
| 11-15 | 829 | 3908 | 252 | 6.45% | 30.40% | 169 | 729 | 51 | 7.00% | 30.18% |
| 16-20 | 119 | 3908 | 20 | 0.51% | 16.81% | 16 | 729 | 4 | 0.55% | 25.00% |
| 21-25 | 109 | 3908 | 29 | 0.74% | 26.61% | 31 | 729 | 10 | 1.37% | 32.26% |
| 26-30 | 230 | 3908 | 51 | 1.31% | 22.17% | 58 | 729 | 11 | 1.51% | 18.97% |
| 31-35 | 298 | 3908 | 95 | 2.43% | 31.88% | 105 | 729 | 24 | 3.29% | 22.86% |
| 36-40 | 180 | 3908 | 46 | 1.18% | 25.56% | 66 | 729 | 14 | 1.92% | 21.21% |
| 41-45 | 87 | 3908 | 18 | 0.46% | 20.69% | 47 | 729 | 2 | 0.27% | 4.26% |
| 46-50 | 77 | 3908 | 6 | 0.15% | 7.79% | 45 | 729 | 4 | 0.55% | 8.89% |
| 51-55 | 130 | 3908 | 20 | 0.51% | 15.38% | 82 | 729 | 7 | 0.96% | 8.54% |
| 56-60 | 138 | 3908 | 23 | 0.59% | 16.67% | 114 | 729 | 10 | 1.37% | 8.77% |
| 61-65 | 154 | 3908 | 17 | 0.44% | 11.04% | 108 | 729 | 4 | 0.55% | 3.70% |
| 66-70 | 201 | 3908 | 13 | 0.33% | 6.47% | 137 | 729 | 2 | 0.27% | 1.46% |
| 71-75 | 213 | 3908 | 12 | 0.31% | 5.63% | 131 | 729 | 2 | 0.27% | 1.53% |
| 76-80 | 191 | 3908 | 12 | 0.31% | 6.28% | 160 | 729 | 3 | 0.41% | 1.88% |
| >80 | 401 | 3908 | 16 | 0.41% | 3.99% | 242 | 729 | 5 | 0.69% | 2.07% |

| Supplementary Table 3 Gender difference of MP infection in different years. | | | | | | |
| --- | --- | --- | --- | --- | --- | --- |
|  | male | | | female | | |
| year | total | pos | pos. rate | total | pos | pos. rate |
|  | 13575 | 3140 | 23.13% | 12405 | 2880 | 23.22% |
| 2018- | 879 | 114 | 12.97% | 712 | 101 | 14.19% |
| 2019 | 1677 | 385 | 22.96% | 1447 | 366 | 25.29% |
| 2020 | 519 | 10 | 1.93% | 453 | 6 | 1.32% |
| 2021 | 850 | 35 | 4.12% | 710 | 30 | 4.23% |
| 2022 | 1086 | 183 | 16.85% | 922 | 153 | 16.59% |
| 2023 | 6273 | 2035 | 32.44% | 6046 | 1873 | 30.98% |
| 2024- | 2291 | 378 | 16.50% | 2115 | 351 | 16.60% |
